# Metabolic landscape of the male mouse gut identifies different niches determined by microbial activities

**DOI:** 10.1038/s42255-023-00802-1

**Published:** 2023-05-22

**Authors:** Karin H. U. Meier, Julian Trouillon, Hai Li, Melanie Lang, Tobias Fuhrer, Nicola Zamboni, Shinichi Sunagawa, Andrew J. Macpherson, Uwe Sauer

**Affiliations:** 1 Institute of Molecular Systems Biology, ETH Zürich, Zürich, Switzerland; 2 Department for Visceral Surgery and Medicine, Inselspital, Bern University Hospital, University of Bern, Bern, Switzerland; 3 Institute of Microbiology, ETH Zürich, Zürich, Switzerland

**Keywords:** Microbiome, Multicellular systems, Gastrointestinal models, Metabolomics

## Abstract

Distinct niches of the mammalian gut are populated by diverse microbiota, but the contribution of spatial variation to intestinal metabolism remains unclear. Here we present a map of the longitudinal metabolome along the gut of healthy colonized and germ-free male mice. With this map, we reveal a general shift from amino acids in the small intestine to organic acids, vitamins and nucleotides in the large intestine. We compare the metabolic landscapes in colonized versus germ-free mice to disentangle the origin of many metabolites in different niches, which in some cases allows us to infer the underlying processes or identify the producing species. Beyond the known impact of diet on the small intestinal metabolic niche, distinct spatial patterns suggest specific microbial influence on the metabolome in the small intestine. Thus, we present a map of intestinal metabolism and identify metabolite–microbe associations, which provide a basis to connect the spatial occurrence of bioactive compounds to host or microorganism metabolism.

## Abstract (web summary)

Anatomically resolved maps of small molecules along the gut of colonized and germ-free mice reveal distinct spatial patterns throughout the gut. These patterns can, in part, be associated with specific microorganisms producing bioactive compounds.

## Main

The mammalian gut is populated by a wide variety of microorganisms, collectively referred to as the gut microbiota^1,2^. The microbiota contributes to digestion and immune functions, and its disruption is linked to multiple diseases^3,4^. In addition to digestive functions and preventing (or causing) infections, intestinal microorganisms are also a source of bioactive compounds that influence the host and other microorganisms^3^. The small and large intestines are physiologically most distinct and are colonized by conserved compositions of taxa^4,5^. The duodenum receives dietary nutrients, pancreatobiliary and gastric secretions. Regional differences in tissue permeability, absorptive transport proteins, pH, regulatory signalling and immune responses further distinguish the jejunal and ileal subregions within the small intestine^5^. Community profiling in animal models has demonstrated distinct microbiota compositions in the different intestinal regions^4,6,7^. Beyond these longitudinal differences, there is considerable variation with respect to physiology and microbiota composition between the luminal content and intestinal mucus^8^. The latter is a dense matrix, particularly in the colon, that consists of cross-linked mucin glycoproteins separating gut-lining epithelial cells from the lumen and its microbiota^9^. The thick outer colonic mucus layer also represents a habitat and nutrient source for microorganisms specialized in the breakdown of mucin^8,10^. Increasing insights into the functional landscape of the digestive tract are exemplified by Clostridia that populate and degrade intestinal mucus, certain Firmicutes that produce characteristic short-chain fatty acids or amino acid derivatives in the large intestine, or specific vitamin-producing bacteria in various intestinal sites^3,11–15^.

Biogeographic differences in taxonomic composition and metabolic activity cannot be assessed using faecal samples because they likely restrict the explanatory power, within limits, to the distal colon^4,16^. This is particularly true for the small intestine, where diet influx, rapid transition times and the secretion of digestive enzymes and antimicrobials dominate intestinal processes and shape the microbiome^6,17,18^. To move towards causality, detailed information about bacterial populations and their metabolic processes in different intestinal subregions can help identify the origin of metabolites. The majority of diet components are metabolized and absorbed in the small intestine, leaving fibres and xenobiotics as well as a small fraction of ingested protein and lipids available for the proximal colonic microbiota^17^. Thus, much of the microbial activity is not apparent from faecal samples, including many of the bioactive compounds of microbial origin that are also present in the small intestine^19^. Analysis of microbial activity in the small intestine of humans requires surgery, very extensive peroral intubation or purging before endoscopy. Consequently, animal models are used to investigate the intestine in its entirety^20,21^. A recent example reported metabolite concentrations along the length of the gut in colonized mice, discovering new microbiota-derived bile acid conjugates that affect the chemistry of all organs, establishing a causal link between microorganisms and their effect on the host^7^. Given the large number of different microorganisms in the various intestinal niches and their diverse metabolic activities, these newly discovered bile acids are just one example illustrating the enormous space of metabolite-based host–microbe and microbe–microbe interactions that await to be unravelled.

Here, we characterize the local intestinal metabolism and community composition in luminal content and mucus from 15 sites along the gut of healthy male specific pathogen-free (SPF) and germ-free mice. In addition to characterizing the four major intestinal habitats of luminal content and mucus in the small and large intestines, this metabolic landscape revealed previously unconsidered metabolic processes within the small intestine. Furthermore, we identified 35 intestinal compounds as microorganism-derived metabolites representing a basis for studies on niche-specific intestinal metabolism.

## Results

### Biogeography of the intestinal metabolome in colonized mice

**Extended Data Fig. 1:**
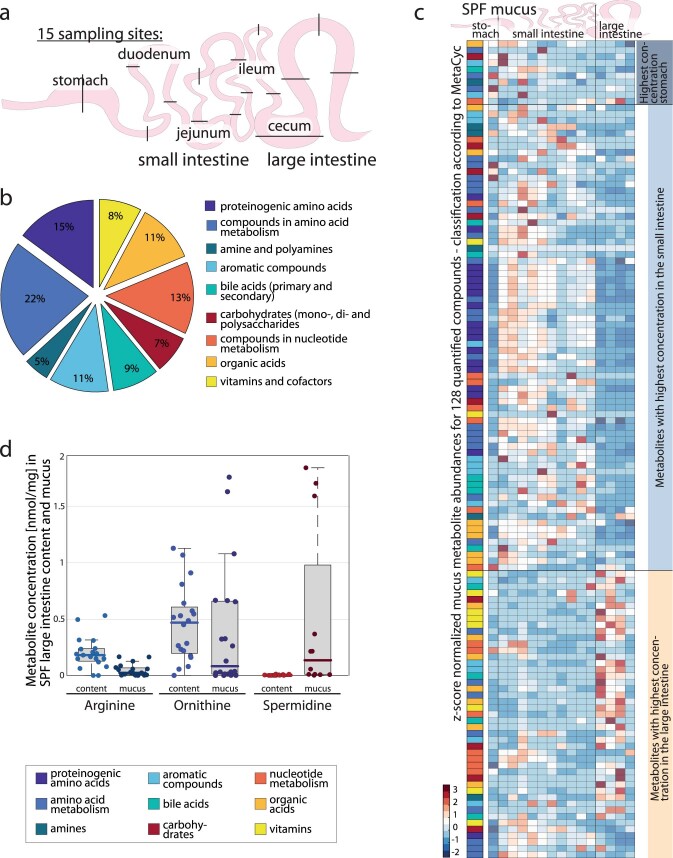
Biogeography of male SPF mice. **a** – Schematic outlining the 15 sampling sites used in this study: one site in the stomach, 10 sites in the small intestine (three in the duodenum, four in the jejunum and three in the ileum), and four sites in the large intestine (in the cecum and in the ascending, transverse and descending colon). **b** – Repartition of MetaCyc chemical classes among all 128 metabolites measured with our LC-TOF-MS method used in this study (Supplementary Table ST1). **c** – Hierarchical clustering analysis of metabolite abundances from intestinal mucus of male SPF mice. Abundances for all 128 quantified metabolites are shown as z-scores of normalized concentrations averaged from five mice, across the 15 sampling sites. The clustering was performed based on Euclidian distances on luminal samples (Fig. 1a) and resulted in the identification of three main clusters, corresponding to the three main known physiological regions of the digestive tract, namely stomach, small and large intestine. In this heatmap here, metabolites along the y-axis are sorted according to the order from Fig. 1a, for comparison. Metabolites in the three main clusters are color-coded according to MetaCyc (as in S1b; Supplementary Table ST1). **d** – Metabolite concentrations of arginine, ornithine and spermidine in SPF luminal content and mucus large intestinal samples. Plotted are 20 data points (four large intestine sites, five male mice), boxplots show median metabolite concentration with a thicker mark, 25^th^ and 75^th^ percentile are denoted by the box, and whiskers extend to the most extreme data points not considered outliers. Source data

To systematically chart metabolome composition along the intestinal tract, we sampled the entire gut of five male SPF C57BL/6J mice at 15 discrete locations (Extended Data Fig. 1a) and separated the luminal contents from the intestinal mucus. To minimize biological variability, 10–14-week-old mice received a standard laboratory diet ad libitum and were fasted for 4 h before sampling, such that the food bulk reached the caecum^21^. Each metabolome sample contained intracellular microbial metabolites as well as extracellular metabolites derived from diet, mouse or microbial activity, and was therefore a complex mixture of chemical compound classes at high salt concentrations. To quantify these gut-relevant compounds, we adopted a liquid chromatography time-of-flight mass spectrometry (LC–TOF–MS) workflow^22^. Briefly, we optimized the method’s run time and established a mix of analytical standards to quantify 138 metabolites that are characteristic of the intestinal metabolome, including compounds in amino acid and nucleotide metabolism, sugars and other carbon sources, bile acids and fermentation products (Extended Data Fig. 1b and Supplementary Table 1). Of these 138 metabolites, 128 could be reliably quantified based on quality control parameters (Supplementary Table 1 and Supplementary Data). The average concentration in the SPF intestinal lumen of these metabolites was approximately 290 nmol mg^−1^ sample in the small intestine and 1.5 µmol mg^−1^ in the large intestine.

**Fig. 1:**
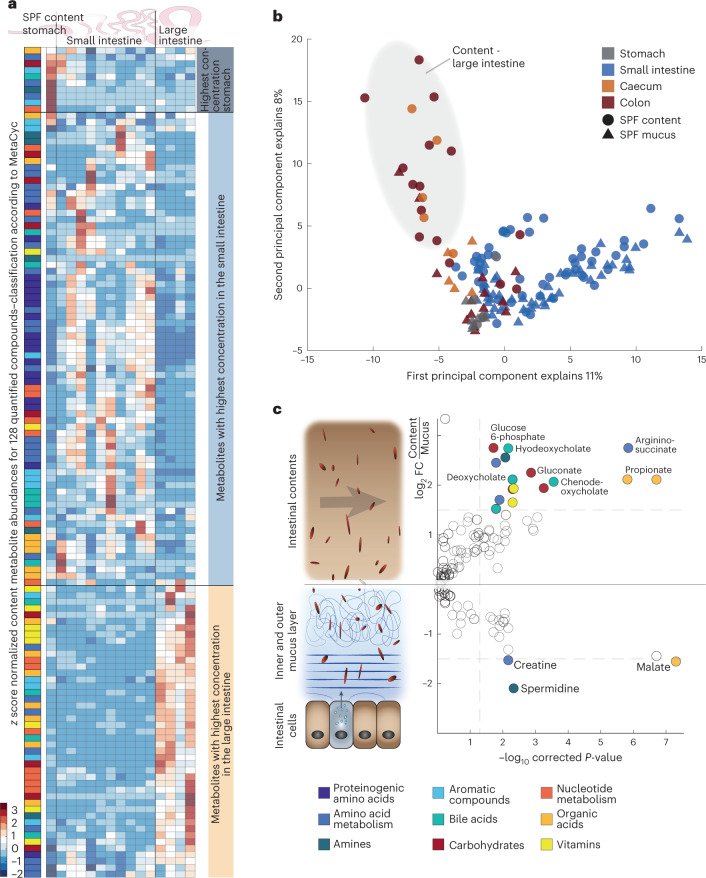
Biogeography of the male SPF mouse metabolome. **a**, Hierarchical clustering analysis of metabolite abundances from luminal content samples of male SPF mice. Abundances for all 128 quantified metabolites are shown as *z* score normalized concentrations, averaged from five mice, across the 15 sampling sites. Clustering based on Euclidian distance identified three main clusters corresponding to the three main physiological regions of the digestive tract: stomach, small intestine and large intestine. Metabolites are colour-coded according to MetaCyc (bottom right). **b**, PCA of metabolite concentrations from individual male SPF mice based on all 150 luminal and mucus samples covering the entire intestine. PCA was performed on *z* score normalized metabolite concentrations, with five mice per sample. Colours indicate sites grouped by intestinal region, symbols indicate lumen or mucus and the large intestine content cluster is highlighted in grey. **c**, Differential analysis of metabolite concentrations between luminal content and mucus samples. Concentrations were averaged across all 15 sampling sites for five male mice within the respective habitat. Positive or negative fold changes indicate higher concentrations in lumen or mucus, respectively. *P* values were calculated using a two-sided paired-sample Student’s *t*-test with Benjamini–Hochberg correction for multiple testing and are displayed as −log_10_ transformed. Metabolites with significantly differing concentrations (absolute log_2_(fold change) ≥ 1.5, corrected *P* ≤ 0.05) are coloured according to the MetaCyc classification, as defined in the box below. Lumen and mucus sampling types are represented schematically on the left, aligned to the corresponding parts of the volcano plot. Abbreviations: FC, fold change. Source data

Spatially resolved analysis of SPF luminal content and mucus allowed us to characterize the heterogeneous metabolic landscape of the mouse intestine. Metabolite profiles over 15 sites were clustered hierarchically for content (Fig. 1a) and, to facilitate comparison, mucus profiles were sorted according to the content clusters (Extended Data Fig. 1c). Bile acids, amino acids and their derivatives were detectable in all sites but had distinct spatial patterns with three main sites of maximal concentrations: stomach, small intestine and large intestine. Stomach contents were characterized by high concentrations of mainly amino acids (Fig. 1a). Proteinogenic amino acids and their derivatives were found almost exclusively in the small intestine. The large intestinal cluster was dominated by organic acids, vitamins and compounds in nucleotide metabolism. Principal component analysis (PCA) separated the small and large intestine, mainly driven by amino acids and their derivatives along the first principal component, corroborating the global differences between the two regions (Fig. 1b). Within the large intestine, the second principal component further separated the metabolome profiles of the luminal content from mucus. Overall, metabolite concentrations in the luminal content were on average 1.7-fold higher than in mucus with bile acids, carbon sources, amino acid derivatives and organic acids as drivers of the separation (Fig. 1c and Supplementary Table 2). Only spermidine, creatine and malate were significantly more abundant in mucus than in luminal samples (*P* ≤ 0.05, fold change ≥3). High spermidine concentrations were presumably a consequence of microbial polyamine production, which is important in maintaining a healthy mucus layer, particularly in the large intestine^23^ (Fig. 1c and Extended Data Fig. 1d). In line with higher polyamine concentrations, the concentrations of the polyamine precursors arginine and ornithine were lower in large intestinal mucus than in luminal content (Extended Data Fig. 1d and Supplementary Table 2).

**Fig. 2:**
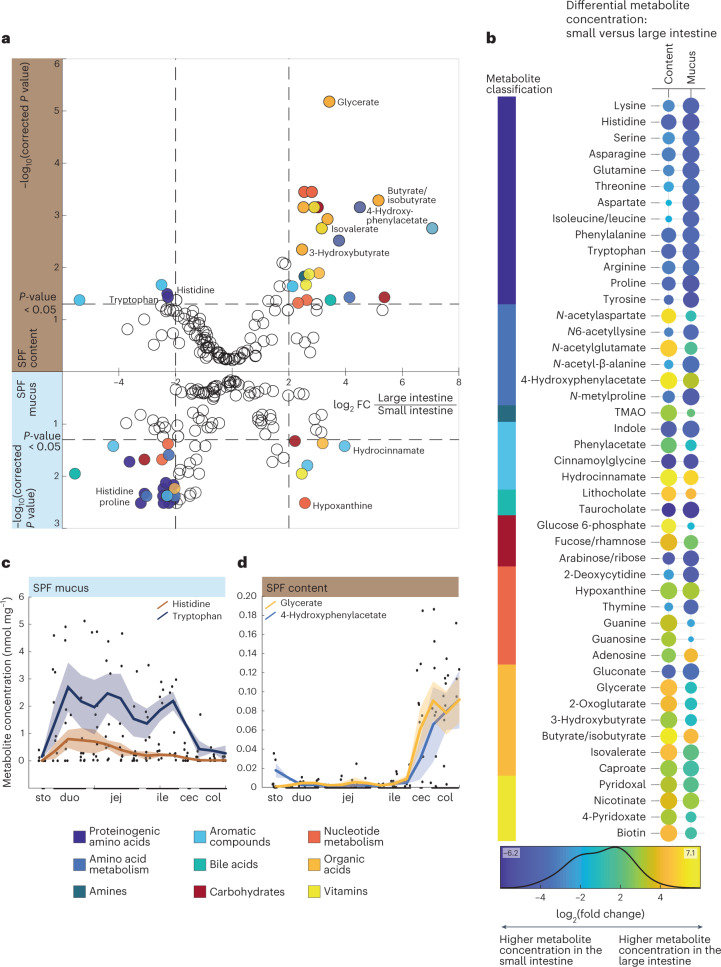
Metabolome differences between small and large intestine of male SPF mice. **a**, Differential analysis of metabolite concentrations between small and large intestinal samples in lumen (upper) and mucus (lower). Data points represent mean fold change values calculated between ten small intestinal and four large intestinal sites, for five male mice. Negative and positive values represent higher concentrations in the small or large intestine, respectively. The *y* axis displays −log_10_-transformed *P* values, calculated using a two-sided paired-sample Student’s *t*-test with Benjamini–Hochberg correction for multiple testing. Metabolites with significantly different concentrations (absolute log_2_(fold change) ≥ 2, corrected *P* ≤ 0.05) are colour-coded according to the MetaCyc classification, as defined in the box (bottom left). **b**, Significantly changing metabolites between the small and large intestine in the lumen and mucus from differential analysis in Fig. 1a (absolute log_2_(fold change) ≥ 2, corrected *P* ≤ 0.05). *P* values were calculated using a two-sided paired-sample Student’s *t*-test with Benjamini–Hochberg correction for multiple testing. Dot colours denote fold change and dot size denotes significance. Metabolites are classified according to MetaCyc, as defined in the box (bottom left). **c**,**d**, Spatial profiles of histidine and tryptophan (**c**) and glycerate and 4-hydroxyphenylacetate (**d**) over 15 intestinal sites in SPF mucus or lumen, respectively. Lines with shaded areas indicate the moving average of the mean ± s.e.m. of concentration measurements from five male mice. Abbreviations: cec, caecum; col, colon; duo, duodenum; ile, ileum; jej, jejunum; sto, stomach; TMAO, trimethylamine *N*-oxide. Source data

To identify specific metabolites that distinguish the small intestine from the large intestine, we calculated fold changes from mean metabolite concentrations in the small and large intestine, separately for luminal content and mucus, across sampling sites and individuals. This differential analysis revealed high concentrations of amino acids and related metabolites in the small intestine, particularly in the mucus (Fig. 2a,b and Supplementary Table 3). Given the rather thin mucus layer in the small intestine, we cannot exclude that the higher proteinogenic amino acid concentrations may, at least in part, be caused by carryover from the lumen during sampling. Representative examples were histidine and tryptophan, the concentrations of which were consistently higher throughout the small intestine and dropped immediately in the large intestine (Fig. 2c). The large intestine was characterized by higher concentrations of organic acids, vitamins and compounds in amino acid and nucleotide metabolism (Fig. 2a,b). Overall, 31 metabolites had at least fourfold higher concentrations in the luminal content or mucus of the large intestine with its higher bacterial load compared with the small intestine (Supplementary Table 3), indicating microbial activity. The sharp increase in short-chain fatty acids such as butyrate/isobutyrate, sugar breakdown products like glycerate and amino acid degradation products like 4-hydroxyphenylacetate indicated extensive microbial fermentation (Fig. 2d). Even though PCA did not distinguish regions within the intestinal mucus (Fig. 1b), differential analysis revealed a distinct metabolic shift from amino acids to fermentation products in mucus (Fig. 2a (bottom), Fig. 2b (right) and Fig. 2c), providing evidence for metabolic differences between the small and the large intestinal mucus habitats.

**Fig. 3:**
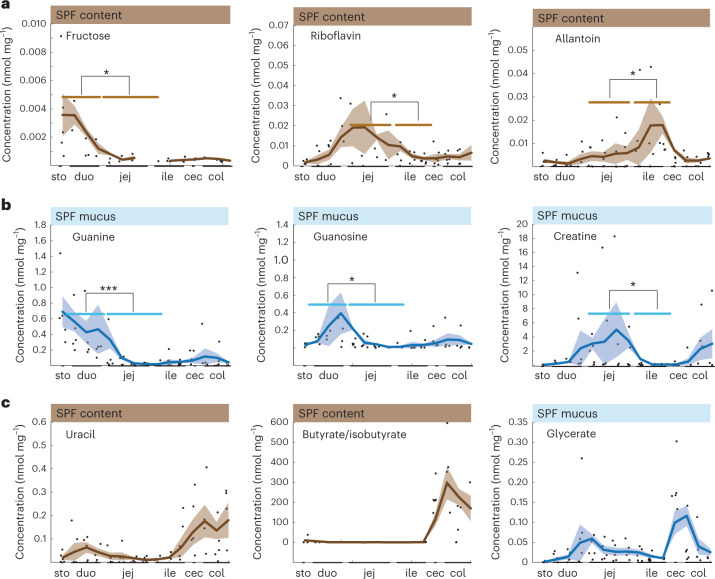
Longitudinal metabolite pattern along the intestine of male SPF mice. **a**–**c**, Example profiles of metabolites with differential concentration in the small intestinal content (**a**) and mucus (**b**), or in the luminal content or mucus of the large intestine (**c**). Lines with shaded areas indicate the moving average of the mean ± s.e.m. of concentration measurements from five male mice. Significantly different metabolite concentrations from one region in the small intestine compared with the neighbouring region are marked by asterisks (*P ≤ 0.05, **P ≤ 0.01, ***P ≤ 0.001). Fructose intestinal content concentration in the duodenum versus jejunum: *P* = 0.0245; riboflavin intestinal content concentrations in the jejunum versus ileum: *P* = 0.0432; allantoin intestinal content concentrations in the jejunum versus ileum: *P* = 0.0220; guanine mucus concentrations in the duodenum versus jejunum: *P* = 0.0005; guanosine mucus concentrations in the duodenum versus jejunum: *P* = 0.0129; creatine mucus concentrations in the jejunum versus ileum: *P* = 0.0278. *P* values were calculated using a two-sided paired-sample Student’s *t*-test. Abbreviations: sto, stomach; duo, duodenum; jej, jejunum; ile, ileum; cec, caecum; and col, colon. Source data

**Extended Data Fig. 2:**
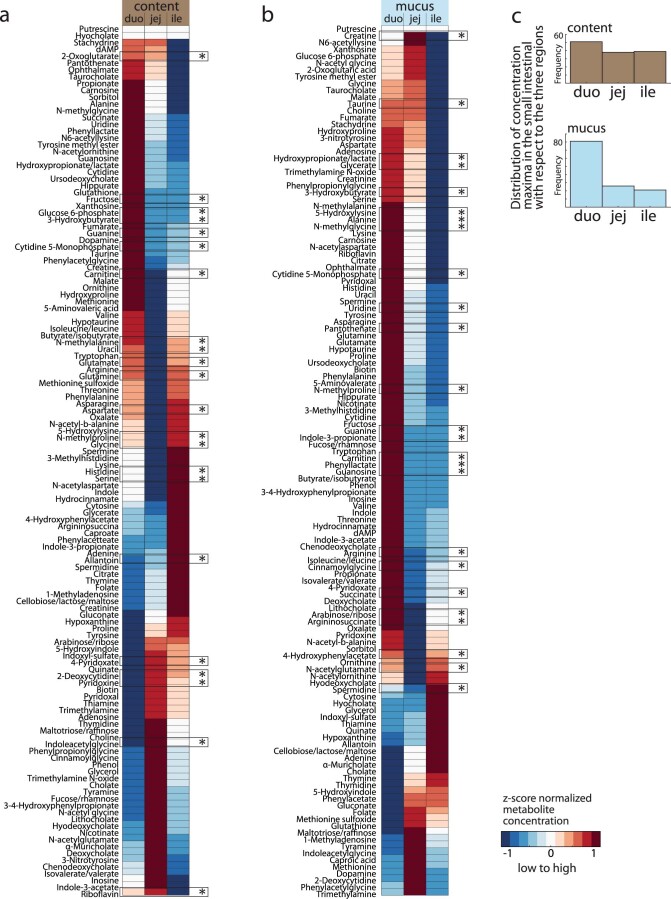
Hierarchical clustering of male SPF small intestinal samples. **a, b** – Hierarchical clustering analysis of metabolite abundances from 10 small intestinal luminal content (**a**) and mucus (**b**) samples of male SPF mice. Abundances of 128 metabolites are shown as z-scores of normalized concentrations averaged from five male mice and three duodenum, four jejunum or three ileum samples, respectively. Hierarchical clustering was performed based on these three averaged values (duodenum, jejunum, ileum). Significantly changing metabolites are marked with boxes and asterisks (p-value ≤ 0.05). P-values were calculated using a two-sided paired-sample Student’s t-test and can be found in Supplementary Table ST4. Color key of the heatmap indicates low to high relative abundance of metabolites throughout the small intestine. **c** – Distribution of peak concentrations in luminal content (top) and mucus (bottom) small intestine, with respect to the three regions. Three bars denote the three regions duodenum, jejunum and ileum, y-axis shows frequency of peak metabolite concentrations occurring in the respective region. Abbreviations: duo – duodenum, jej – jejunum, and ile – ileum. Source data

**Extended Data Fig. 3:**
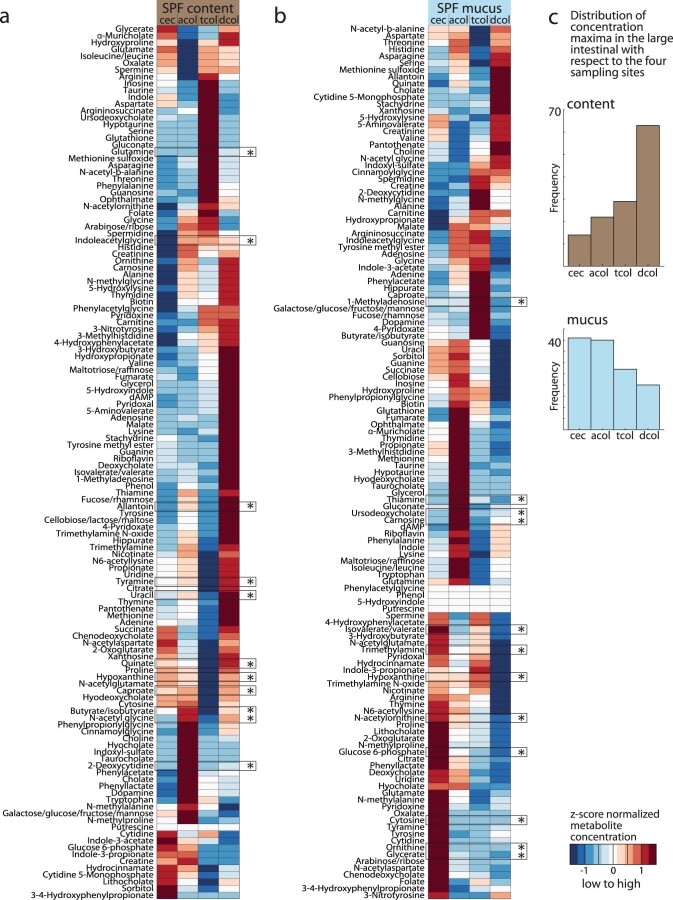
Hierarchical clustering of male SPF large intestinal samples. **a, b** – Hierarchical clustering analysis of metabolite abundances from four large intestinal luminal content (**a**) and mucus (**b**) samples in male SPF mice. Abundances of 128 metabolites are shown as z-scores of normalized concentrations averaged from five male mice per sampling site in the colon (cecum, ascending, transverse and descending colon). Hierarchical clustering was performed only on these four values. Significantly changing metabolites are marked with boxes and asterisks (p-value ≤ 0.05). P-values were calculated using a two-sided paired-sample Student’s t-test. Color key of the heatmap indicates low to high relative abundance of metabolites throughout the large intestine. **c** – Distribution of peak concentrations in luminal content (top) and mucus (bottom) large intestine, with respect to the four regions is depicted. Four bars denote the regions, y-axis shows frequency of peak metabolite concentrations occurring in the respective region. Abbreviations: cec – cecum, acol – ascending colon, tcol – transverse colon, and dcol – descending colon. Source data

The overall metabolome pattern did not allow us to distinguish individual sites within the small or large intestines because differences between individual mice at these sites were, expectedly^24^, just as large (Fig. 1b), but about one-third of the metabolites exhibited distinct longitudinal profiles (Fig. 3 and Supplementary Table 4). Hierarchical clustering of metabolite concentrations in the small intestine resulted in three groups: metabolites with a maximum concentration in the duodenum, the jejunum or the ileum (Extended Data Fig. 2). Although the three classes were distributed equally in luminal samples, most mucus concentrations decreased towards the ileum in the small intestine samples (Extended Data Fig. 2b,c). Metabolites with high concentrations in the duodenum content included fructose (Fig. 3a) and other monosaccharides, presumably residual dietary components that had been absorbed by the host before reaching the distal small intestine^14,25^. Riboflavin concentrations were highest in the jejunum content, originating either from microbial production or residual dietary vitamins^14,15,26^. High allantoin concentrations in the ileal content (Fig. 3a) probably resulted from microbial degradation of purine metabolites in the upper small intestine of coprophagic mice^27^, consistent with high duodenum mucus concentrations of guanine and guanosine (Fig. 3b) and the major influx of guanine and guanosine through the chow diet. High creatine concentrations in jejunal mucus may be the result of dietary creatine, whereas the concentration decrease was probably due to uptake by high energy requiring intestinal cells that express various creatine transporters and creatine-metabolizing enzymes^28^. In the large intestine, hierarchical clustering revealed maximum luminal content concentrations mostly in the distal colon, whereas mucus peak concentrations were more evenly distributed throughout the large intestine (Extended Data Fig. 3). In luminal content, uracil concentrations were highest in the distal colon, whereas butyrate/isobutyrate appeared to decrease slightly towards the distal colon (Fig. 3c) where butyrate is the major energy source of colonocytes^29^. In large intestinal mucus, the fructose breakdown product glycerate^25^ peaked in the caecum but dropped sharply in the colon. Altogether, our data suggest microbial activity as a possible factor contributing to the longitudinal concentration profiles of 42 small intestinal metabolites.

This map of intestinal metabolism in SPF mice highlights the luminal content and mucus of the small and large intestine as main niches with a largely consistent metabolome pattern throughout multiple sampling sites. The general shift from higher concentrations of amino acids and their derivatives in the small intestine to vitamins and fermentation products in the large intestine is presumably caused by greater microbial activity in the large intestine, indicating a microbial origin for 31 metabolites. Moreover, we quantified substantial patterns within the small intestine for 42 of the 128 measured metabolites. Because these metabolic differences could, at least in part, result from microbial activity in different regions of the gut, we next investigated the role of the microbiota.

### Microbiota influence the intestinal metabolic landscape

**Extended Data Fig. 4:**
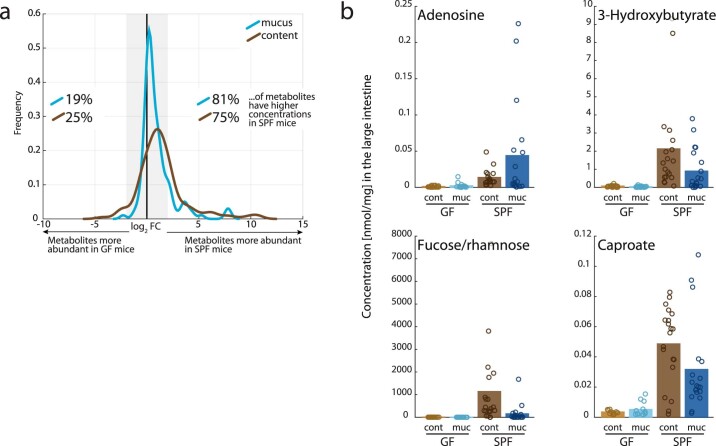
Microbiota effect on large intestinal metabolism. **a** – Distribution of metabolite concentration fold changes in the SPF versus germ-free comparison in luminal content and mucus samples. Fold changes are calculated per metabolite in the respective habitat for five male mice and 15 sampling sites, log_2_ transformed, and their distribution is displayed as contour plot along the x-axis. The frequency is denoted on the y-axis. A negative log_2_ fold change indicates higher metabolite concentration in germ-free mice, a positive log_2_ fold change indicates higher concentration in male SPF mice. In grey, all absolute fold changes ≤ 2 are marked. **b** – Large intestinal concentration of adenosine, 3-hydroxybutyrate, fucose/rhamnose, and caproate in SPF and germ-free luminal content and mucus. Solid bars show mean concentration of measurements from five male mice, averaged over the four large intestinal sites. The 20 corresponding data points are displayed as circles. Abbreviations: FC – fold change; GF – germ-free; duo – duodenum, jej – jejunum, and ile – ileum; cont – luminal content; muc – mucus. Source data

High metabolite concentrations in the large intestine (Fig. 2a), known for its extensive microbial activity^13,14,29^, and the spatial metabolite pattern in the small intestine (Fig. 3) provided only indirect evidence for a microbial origin. Besides being microorganism-derived, these metabolites could also be host metabolites produced as a response to microorganisms, residual dietary components or intracellular metabolites from shed intestinal cells. To obtain more direct evidence for microbial influence on intestinal metabolism and to dissect longitudinal changes in the affected metabolites, we performed a matching experiment with five male germ-free C57BL/6J mice, whose intestinal contents and mucus were sampled at the same 15 sites after fasting. Generally, at least two-thirds of the metabolites had higher concentrations in the presence of bacteria; nearly 50% of luminal content and 30% of mucus metabolites had at least twofold higher concentrations in SPF mice than in germ-free mice (Extended Data Fig. 4a and Supplementary Tables 5 and 6). Metabolites with particularly high concentrations in SPF mice included bile acids, organic acids, aromatic compounds, vitamins and amino acid derivatives (Supplementary Tables 5 and 6). Only about one-quarter of the metabolites were more abundant in germ-free mice, mainly carbon sources and compounds of nucleotide as well as amino acid metabolism that might be of dietary origin and are depleted by the microbiota in SPF mice^30^.

**Fig. 4:**
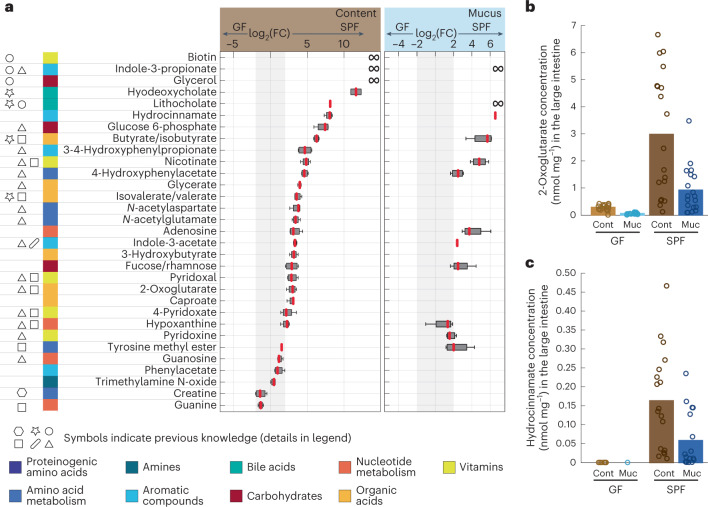
Microbiota effect on large intestinal metabolism. **a**, Abundance fold changes of 31 metabolites with higher large intestine than small intestine concentrations, thus hypothesized to be of microbial origin (significantly changing metabolites from differential analysis; Fig. 2a,b). The log_2_-transformed fold changes for luminal content (left) and mucus (right) were calculated from averaged concentrations for the four large intestinal sampling sites of five male SPF mice versus five male germ-free mice. Boxplots are thus based on 20 data points, the median log_2_(fold change) is indicated in red, boxes contain the 25th to 75th percentiles, and the whiskers extend to the most extreme data points not considered outliers. The grey area marks absolute log_2_(fold change) ≤ 2. Symbols on the left indicate previous evidence: () common knowledge about certain metabolites, for example see Koh et al. and de Aguiar Vallim et al.^29,66^; (Δ) evidence from Han et al.^19^ using the comparison of Swiss-Webster germ-free versus conventional mice to identify differential metabolite abundances; (□) evidence from Matsumoto et al.^33^ using germ-free and ‘ex-germ-free’ mice that were inoculated in the stomach with faecal suspension from SPF BALB/c mice to classify metabolites as mouse- or microorganism-derived; () evidence from Marcobal et al.^30^ who used germ-free and conventional Swiss-Webster mice to compare metabolite levels; () evidence from Sridharan et al.^32^ using reaction network models to predict microbiota-dependent metabolic products. All data used from published work including explanations can be found in Supplementary Table 7. In addition, (◯) denotes metabolites detected exclusively in male SPF mice. For the SPF exclusive metabolites, fold changes cannot be calculated, as indicated by ∞. **b**,**c**, Large intestinal concentration of 2-oxoglutarate (**b**) and hydrocinnamate (**c**) in SPF and germ-free luminal content and mucus. Solid bars show the mean concentration of measurements from five male mice, averaged over the four large intestinal sites. The 20 corresponding data points are displayed as circles. Abbreviations: FC, fold change; cont, luminal content; GF, germ-free; muc, mucus. Source data

Among the above 31 metabolites of potential microbial origin, 24 had at least fourfold higher mean concentrations in the large intestine of SPF mice compared with germ-free mice (Fig. 4a and Supplementary Table 7). The short-chain fatty acids butyrate/isobutyrate and isovalerate/valerate, and the secondary bile acids lithocholate and hyodeoxycholate had at least 14-fold higher concentrations in the large intestine of SPF mice. Such high concentrations are consistent with their well-known formation by the abundant low oxygen and low pH tolerating Bacteroidales in the colon^4,31^. Biotin, indole-3-propionate and glycerol were detected exclusively in SPF mice, providing strong evidence for their microbial origin. Indole-3-propionate is a well-established gut microbiota-derived compound resulting from tryptophan metabolism^13,32^. For most of the 31 large intestinal metabolites, our hypothesis of microbial origin was consistent with either targeted studies or previously observed higher levels in colonized mice than in germ-free mice^3,19,30,32,33^ (Fig. 4a and Supplementary Table 7). The higher SPF concentration of the ubiquitous intracellular metabolite 2-oxoglutarate (Fig. 4b) is most likely explained by the high bacterial load, as described previously^34^. Newly identified metabolites of microbial origin were hydrocinnamate, 3-hydroxybutyrate, caproate, adenosine and fucose/rhamnose (Fig. 4c and Extended Data Fig. 4b). Akin to phenylacetate and 4-hydroxyphenylacetate, hydrocinnamate may result from the fermentation of aromatic amino acids, as shown in vitro for several gut microorganisms^35^. Likewise, 3-hydroxybutyrate and caproate are fermentation products^36^. Sugars such as fucose/rhamnose are most likely liberated from dietary fibres though secreted microbial glycoside hydrolases. Consistent with previous observations^19,30^, the higher large intestinal concentrations of creatine and guanosine in germ-free mice suggest a host or diet origin (Fig. 4a). Altogether, higher metabolite concentrations in SPF mice than in germ-free mice thus provide supporting evidence for the microbial origin or microbiota-induced host production of 24 of the 31 metabolites with high large intestinal concentrations.

To rule out dietary contributions to elevated metabolite concentrations, we determined the composition of chow diet pellets using untargeted flow-injection analysis time-of-flight mass spectrometry (FIA–TOF–MS)^37^. In brief, we dissolved and extracted three food pellets according to the protocol used for intestinal samples. Based on accurate mass and isotopic patterns, we putatively annotated 1,379 metabolite ions. Despite differences in ionization efficiencies, the measured intensities allowed us to roughly estimate the relative abundance of the detected compounds. The distribution of apparent abundances was remarkably skewed. Seventeen metabolites made up 50% of the total signal recorded in pellets (Supplementary Table 9), with disaccharides being the dominant contributor (14% on average). Other abundant metabolites were adenine, malate, hexoses, lysine, guanosine, guanine and tryptophan. High dietary concentrations of the purine nucleotides guanosine and guanine explain their abundance in germ-free mice and low concentrations in the SPF gut where we observed a concomitant increase in the purine breakdown product allantoin. Most importantly, none of the 24 metabolites that we found to be linked to the microbiota contributed more than 0.05% to the diet composition. Hence, the abundance of these markers in the diet appears to have a minor role.

**Fig. 5:**
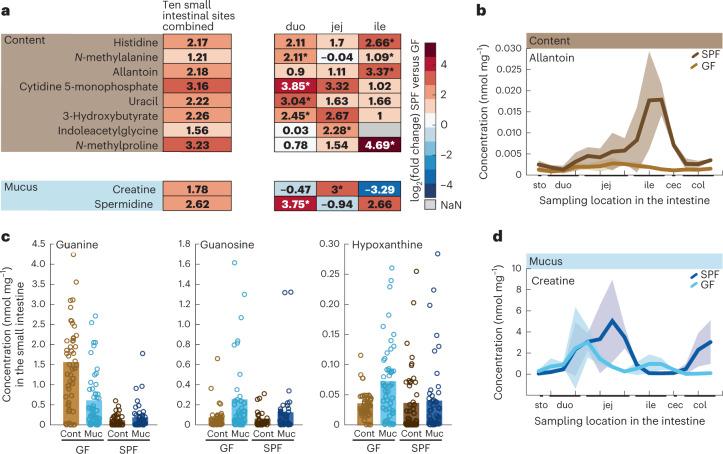
Microbiota effect on small intestinal metabolism. **a**, Heatmap representation of metabolites with at least fourfold higher concentrations in male SPF mice than in male germ-free mice, specifically in one location of the small intestine versus the whole small intestine. The left-hand column shows averaged log_2_-transformed fold changes of all ten small intestinal sites (SPF versus germ-free), and the next three columns depict averaged fold changes (SPF versus germ-free) in the duodenum, jejunum or ileum separately. From spatial profiles (as in Fig. 3, not all shown here), we identified the region (duodenum, jejunum or ileum) that varies from its neighbouring region. For that region, marked by an asterisk, we suspect microbial involvement causing the distinct difference, and consequently also a higher SPF over germ-free concentration. **b**, Spatial profiles of allantoin concentrations in SPF and germ-free luminal content. Lines with a shaded area indicate the moving average of the mean ± s.e.m. of concentration measurements from five male mice. **c**, Small intestine concentrations of guanine, guanosine and hypoxanthine in SPF and germ-free luminal content and mucus. Solid bars show the mean concentration of measurements from five male mice, over ten small intestinal sites. The 50 corresponding data points are displayed as circles. **d**, Spatial profiles of creatine concentrations in SPF and germ-free mucus. Lines with a shaded area indicate the moving average of the mean ± s.e.m. of concentration measurements with five male mice. Abbreviations: FC, fold change; GF, germ-free; vs., versus; sto, stomach; duo, duodenum; jej, jejunum; ile, ileum; cec, caecum; col, colon; cont – luminal content; muc – mucus; NaN, not a number (fold changes could not be calculated). Source data

**Extended Data Fig. 5:**
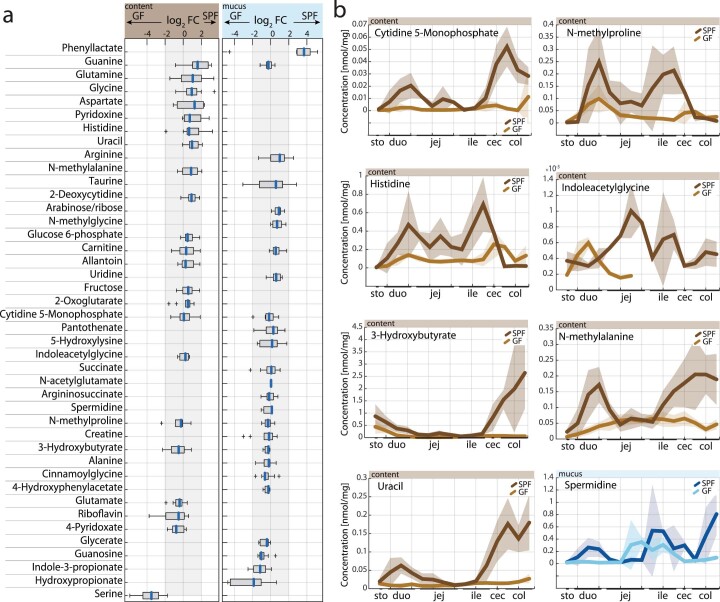
Small intestinal metabolism. **a** – Log_2_ transformed fold changes of the mean small intestinal concentration of 42 metabolites with longitudinal patterns in the small intestine (see Fig. 3 for example profiles, Fig. 5, Supplementary Table ST4). Fold changes were calculated between male SPF and male germ-free samples in luminal content (left panel) and mucus (right panel), averaged over the 10 sampling sites and five biological replicates. Boxplots are thus based on 50 data points, where the median log_2_ fold change is indicated in blue, 25^th^ and 75^th^ percentile are denoted by the box, whiskers extend to the most extreme data points not considered outliers, and outliers are marked with the plus symbol. The grey area represents absolute log_2_ fold changes ≤ 2. **b** – Spatial metabolite profiles of luminal content or mucus concentrations in SPF and germ-free mice. Lines with shaded area indicate the moving average of the mean ± s.e.m. of concentration measurements with five male mice. Abbreviations: FC – fold change; GF – germ-free; duo – duodenum, jej – jejunum, ile – ileum, cec – cecum, and col – colon. Source data

Lastly, we looked for evidence of microbial origin among the 42 metabolites with a longitudinal pattern in the small intestine of SPF mice. With the exception of phenyllactate, none of them matched our criterion of a fourfold higher concentration in SPF mice compared with germ-free mice when averaging across all ten sampling sites (Extended Data Fig. 5a). In specific locations within the small intestine, however, eight luminal content and two mucus metabolites reached fourfold higher concentrations in SPF mice than in germ-free mice when considering the duodenum, jejunum and ileum separately (Fig. 5a and Extended Data Fig. 5b,c). For example, concentrations of the purine metabolism end-product allantoin increased throughout the small intestine of SPF mice, reaching a maximum in the ileum, whereas levels remained low in germ-free mice (Fig. 5b). Consistently, concentrations of the dietary purine metabolites guanine, guanosine and hypoxanthine were lower in SPF mice than in germ-free mice (Fig. 5c), providing evidence for their microbial breakdown and subsequent conversion to allantoin. A different profile was seen for creatine, whose concentration increased initially in the mucus of both SPF and germ-free mice but dropped to baseline in germ-free mice while reaching a maximum in the jejunum of SPF mice (Fig. 5d). The initial coinciding concentration increase was possibly due to its presence in the diet, but the differential pattern in the jejunum indicated either microbial production or microbial alteration of creatine consumption by intestinal cells^35^. The other eight metabolites with higher peak concentrations in SPF mice included several amino acid or nucleotide metabolism-related compounds. An example is the aromatic indole-derived indoleacetylglycine, the concentration of which fluctuated throughout the SPF small intestine, but was below the detection limit in the distal small intestine of germ-free mice (Extended Data Fig. 5b), which strongly suggests microbial influence. The consistently higher small intestinal concentrations of cytidine 5-monophosphate, uracil and histidine are best explained as intracellular microbial metabolites. In addition to the 24 metabolites in the large intestine, we thus provide evidence for the microbial origin of 11 metabolites in the small intestine.

### Spatial metabolite patterns can be associated with microorganisms

**Extended Data Fig. 6:**
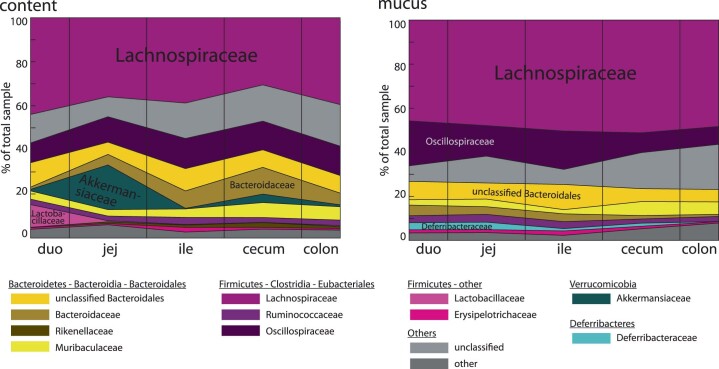
Microbiome composition of male SPF luminal content and mucus. Community composition and differences throughout the gut of male SPF luminal content (upper panel) and mucus (lower panel) over five sites: duodenum, jejunum, ileum, cecum and colon. Displayed are relative microbe abundances in percent, on the family level. The top 95% of annotated community members per sample are displayed discretely, bacterial categories representing less than 5% of the community are grouped in “others”. Source data

To investigate whether the metabolome patterns were associated with the microbiome composition, we profiled luminal content and mucus community composition using 16S ribosomal RNA sequencing of the duodenum, jejunum, ileum, caecum and colon of five cohoused male SPF mice from the same litter. In all five sites, the community was dominated at the phylum level by Firmicutes and at the family level by Lachnospiraceae, Oscillospiraceae, Lactobacillaceae and Bacteroidaceae (Extended Data Fig. 6a). As expected^7^, the relative abundance of Bacteroidales increased in the large intestine, particularly in lumen samples, coinciding with high short-chain fatty acids and secondary bile acid concentrations. Consistent with the metabolome patterns, the community composition was largely invariant across the three small intestinal sites. The main differences between luminal content and mucus with respect to composition were a generally greater diversity and the relative abundance of Akkermansiaceae in the luminal content (Extended Data Fig. 6a). These differences were most pronounced in the jejunum, where up to 20% of the entire content community were Akkermansiaceae.

**Fig. 6:**
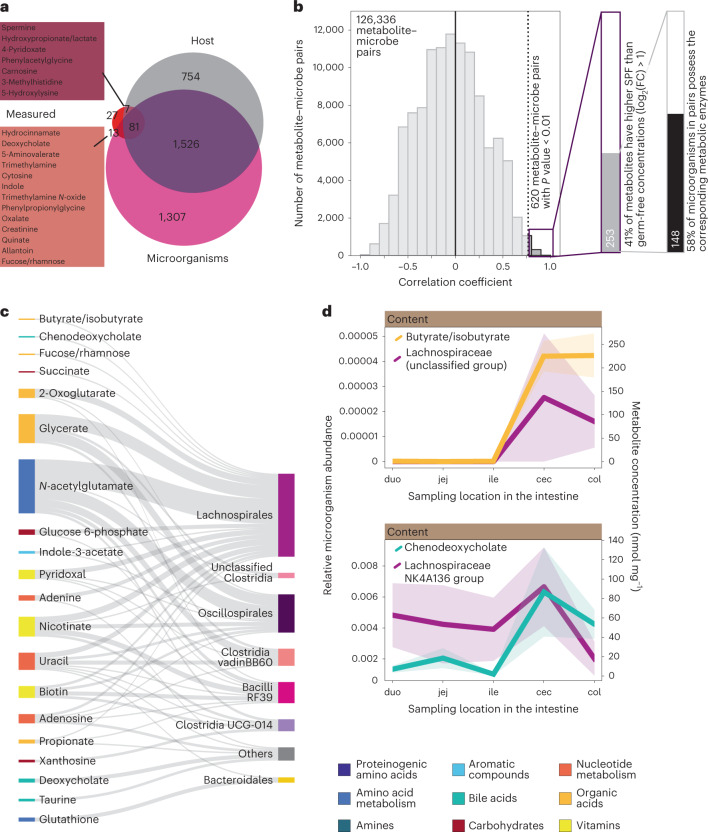
Metabolite–microbe associations. **a**, Venn diagram showing the overlap of 128 measured metabolites classified as host- or microorganism-related, based on PICRUSt2 predictions of microbial metabolic functions and the mouse metabolic network. The majority of metabolites cannot be classified as either host- or microbe-associated, seven metabolites are host-related and thirteen metabolites are microorganism-related. Twenty-seven metabolites did not match any of the metabolic networks. **b**, Distribution of the correlation coefficients of 126,336 metabolite–microbe pairs. Zoomed-in bars show how applying thresholds on the *P* value, the SPF–germ-free fold change and the presence of metabolic enzymes reduces the number of predicted functional metabolite–microbe pairs to 148. **c**, Sankey diagram showing links between unique metabolites and the corresponding microbial orders in positively correlated metabolite–microbe pairs that meet the thresholds defined in **b**. Metabolites are colour-coded according to MetaCyc. The size of the linkage line denotes number of pairs. **d**, Spatial profiles of metabolite concentrations and associated microorganisms in SPF lumen samples. Lines with a shaded area indicate the mean ± s.e.m. of concentration measurements with five male mice, and the mean ± s.e.m. of relative microorganism abundance. Abbreviations: duo, duodenum; jej, jejunum; ile, ileum; cec, cecum and col, colon. Source data

To assess the contribution of microorganisms to the measured gut metabolome, we determined the functional potential of the microbiome using the PICRUSt2 tool^38^ that predicts the enzyme pool of each detected microorganism. By comparing the obtained set of all potential microbial reactions with the known set of metabolic reactions in mice from the Kyoto Encyclopedia of Genes and Genomes (KEGG) database, metabolites were classified as potentially linked to the host (7), the microbiome (13) or both (81) (Fig. 6a). For example, the high concentrations of spermine and hydrocinnamate in SPF mice are best explained by a host and microbiome origin, respectively. To identify the producing species, we correlated metabolite and microorganism abundances spatially along the SPF gut. Overall, correlation coefficients exhibited a normal distribution, with 620 metabolite–microbe pairs showing significant co-occurrence (*P* < 0.01) (Fig. 6b). To refine predictions of potential microbial production, we considered only metabolites that were more abundant in SPF mice than germ-free mice (fold change >2). Based on the above predicted microbial metabolic reactions, we further restricted our analysis to pairs in which the predicted microorganisms possess enzymes that catalyse reactions involving the paired metabolite. Altogether, we predicted 148 pairs of potential microbial metabolite production from the correlation of 20 metabolites with the abundance of 91 microorganisms encompassing 14 different bacterial orders (Fig. 6c and Supplementary Table 10). As expected from their ubiquitous nature, metabolic intermediates such as *n*-acetylglutamate and the fructose breakdown product glycerate were linked to 41 and 22 microorganisms, respectively. Ten metabolites were more specifically linked to three or fewer microorganisms, including a single microorganism link for butyrate, chenodeoxycholate, rhamnose and succinate. For butyrate and chenodeoxycholate, which are the metabolites with the highest SPF versus germ-free fold change, we pinpointed an unclassified group of the known short-chain fatty acid producer Lachnospiraceae^39^ and members of the Lachnospiraceae NK4A136 group as the responsible microorganisms, respectively (Fig. 6d). These and other observed spatial co-occurrences of metabolites with specific microorganisms further strengthen our hypothesized link to microbial activity.

## Discussion

We report an anatomically resolved map of intestinal metabolism in healthy male SPF and germ-free mice, revealing the luminal content and mucus of the small and large intestines as four metabolically distinct niches. The small intestinal metabolome was dominated by amino acids and the large intestine was characterized by high concentrations of fermentation products, vitamins and compounds in nucleotide metabolism, consistent with the much higher density of microorganisms in the large intestine^4^. In line with its physicochemical and immunological characteristics^8,40^, the densely populated outer mucus layer lining the gut was also metabolically distinct with microorganism-derived polyamines and creatine as the most distinguishing features. Creatine and polyamines such as spermidine are known to play key roles in maintaining a healthy mucus layer, especially in the presence of a microbiota^35,41,42^. Exceedingly high concentrations of proteinogenic amino acids distinguished the thin layer of mucus in the small intestine from the thicker layer in the large intestine^4^, possibly a consequence of diffusion from the luminal content or enzyme-mediated protein breakdown.

In addition to characterizing the four major intestinal niches, our dataset resolved the spatial pattern of 42 metabolites within the small intestine, a part of the intestinal tract that is inherently difficult to access^18^ and where microorganisms produce many bioactive compounds^19^. High concentrations of fructose and other monosaccharides in the duodenum, downstream of the stomach, probably resulted from the breakdown of diet components that were later absorbed by the host^14,25,43^. Towards the end of the small intestine, we found evidence of the microbial conversion of purine metabolites leading to high allantoin concentrations in the ileal content. Likewise, high fluctuating concentrations of indole derivatives such as indoleacetylglycine in the small intestine of SPF mice suggested a microbial origin, which has so far been described mainly in the large intestine^44^. Microbial indole metabolism is important because indoles regulate the release of intestinal hormones that control host digestion^45^. Indole derivatives also signal as agonists through the aryl hydrocarbon receptor that regulates epithelial barrier integrity, in consequence limiting intestinal inflammation^6,46^. Our high-resolution metabolome data thus provide insights into microorganism-associated metabolic dynamics within subregions of the small intestine, thereby adding functional information to established resources such as transcriptional data in the Tabula Sapiens project^47^ and other comprehensive studies^7^. Our metabolic variability is generally consistent with the reported microbiome fluctuations between different sampling sites within intestinal subregions, although the functional metabolite data appear to vary much less than the microbial composition, which is likely explained by the functional redundancy of the gut microbiome^48^.

Based on the higher concentration in SPF mice compared with germ-free mice, we provided evidence of a microbial origin for 11 and 24 metabolites in the small and large intestine, respectively. In addition, by predicting the microbiome metabolic potential, and performing spatial co-occurrence analyses, we could identify specific microorganisms as potential producers for 20 metabolites. Although the small intestinal metabolic niche is mainly determined by the diet and the host’s digestive activities^16^, we showed that the gut microbiota contributes to shaping the small intestinal metabolome beyond lipid and bile acid metabolism^6,49^. Our anatomically resolved metabolic map allowed us to infer some of the underlying processes of the metabolite pattern, such as high ileal concentrations of allantoin that appear to result from the microbial breakdown of purines, potentially providing nitrogen in the generally nitrogen-limited environment of the gut^50^. Continuously increasing creatine concentrations in the jejunum of SPF mice contrast with the lower concentrations in germ-free mice, suggesting either microbial production, alteration of creatine consumption by host intestinal cells^35,51^ or stimulation of creatine production in the liver and kidney^28^. The 24 microbiota-derived metabolites in the large intestine include several established microbial products such as the short-chain fatty acids butyrate/isobutyrate and isovalerate/valerate, as well as the microorganism-related secondary bile acids lithocholate and hyodeoxycholate^31,52,53^. Several studies previously reported that different subsets of these compounds are highly abundant in the faeces or colon of colonized mice^3,19,30,32,33^, but we newly identified hydrocinnamate, adenosine, 3-hydroxybutyrate, caproate and fucose/rhamnose as compounds of microbial metabolic origin. Rhamnose and adenosine could be linked to specific Clostridia and Bacilli that contain enzymes necessary for their formation. Co-occurring microorganisms are potential producers of the other three novel compounds, but such correlations may also indirectly result from microbial growth on cross-fed metabolites. By contrast, hydrocinnamate (also known as 3-phenyl-propionate) cannot be produced by eukaryotic cells. Thus, the 259-fold higher concentration in the large intestine of SPF mice compared with germ-free mice presumably results from the conversion of phenylalanine and tyrosine through Clostridia^13^, which coincides with clostridial abundances in the large intestine that make up roughly half of the entire SPF microbiome^4,13,29^. The ketone body 3-hydroxybutyrate is a host metabolite with primary production in the liver, but it is also a potential product of various Clostridia identified in our 16S data. Its tenfold higher concentration in the large intestine of SPF mice may thus be due to microbial secretion, microbial induction of 3-hydroxybutyrate production by the host or a combination of both, the clarification of which would require further experiments.

To our knowledge, we provide here the first anatomical in vivo map of unperturbed lumen and mucus metabolism along the entire mouse intestine. We thereby also consolidate findings from previous studies investigating dietary interventions, disease models or specific intestinal sites. Comparing metabolic landscapes in male colonized SPF versus germ-free mice allowed us to disentangle the origin of many metabolites in different niches, and in some cases to infer the underlying processes. These associations are further strengthened through a correlation analysis pinpointing specific metabolite–microbe associations. Although we lack comparison with faeces, on the basis of these metabolic differences and reported differences in microbiota^7,16^, we conclude that the intestine is a far too variable an environment to be approximated using faecal samples only, as demonstrated for mono-colonized mice^54^. Our dataset also provides a starting point for future studies on gut metabolism.

## Methods

### Mice and sampling

All mouse experiments were performed in accordance with Swiss Federal and Cantonal regulations. Permission was granted by the commission for animal experimentation of the Kanton Bern. Male C57BL/6J mice were bred and maintained at the Clean Mouse Facility, University of Bern, Switzerland. Male colonized SPF mice on a C57BL/6J background were purchased from Envigo. Male germ-free C57BL/6J mice were obtained via caesarean section and maintained with aseptic husbandry within flexible film isolators. Five replicate animals were used per group. All mice were 10–14 weeks of age, confirmed to be pathogen-free and maintained under a 14-h light/10-h dark cycle at an average temperature of 20 °C and 40% humidity. All mice received standard laboratory Kliba Nafag 3436 chow diet and water ad libitum. Mice were killed by cervical dislocation at the same time of day to control for variations due to the circadian rhythm after 4 h of fasting, which allows completion of transit through the small intestine^55^. Residual diet will be in the caecum and replicate mice are thus more comparable. Samples from the luminal contents and the intestinal mucus were collected at 15 sites along the intestine, as described previously^8^. In brief, the entire gastrointestinal tract was resected via an abdominal incision. The different intestinal regions (stomach, small intestine, caecum and colon) were separated, opened longitudinally, the luminal contents were removed and mucus was scraped off the intestinal walls. Samples were flash frozen in liquid nitrogen.

### Metabolite extraction from intestinal samples and food pellets

Metabolites were extracted from 50 mg of sample in 20 volumes per weight 80 °C Millipore water. Samples were vortexed for 10 s, incubated for 3 min at 80 °C in an Eppendorf Thermomixer at maximal speed (1,500 r.p.m.), vortexed for 10 s and centrifuged for 3 min at 20,000*g* at room temperature. From the supernatant, 150 µl were transferred to 96-well microtiter plates and stored at −80 °C. For mass spectrometry analysis, the samples were diluted 1:1 in mobile phase (methanol/water/acetic acid 49.6:49.6:0.8 v/v/v) containing the retention time reference compound 9-anthracene carboxylic acid (Sigma-Aldrich) and stored at −20 °C until analysis. For metabolite extractions from food pellets, three pellets were weighed and dissolved in 20 ml of Millipore water overnight at room temperature. The next day, 10 ml of Millipore water was added and vortexed for 10 s. One millilitre of food pellet suspension was heated to 80 °C and the metabolites were extracted as described above (3 min incubation at 80 °C in an Eppendorf Thermomixer at maximal speed (1,500 r.p.m.), vortexed for 10 s and centrifuged for 3 min at 20,000*g* at room temperature). From the supernatant, 150 µl were transferred to 96-well microtiter plates and stored at −80 °C until analysis. Before measurements, samples were 1:20 diluted in MilliQ water.

### Chemicals

HPLC-grade methanol and isopropanol, all chemicals, buffer additives for online mass referencing and sample preparation chemicals were purchased from Sigma-Aldrich, Agilent Technologies and Cayman Chemicals. HPLC-grade water was obtained using an IQ7000 MilliQ water purification system equipped with an LC-Pak (Merck).

### Metabolite profiling using LC–TOF–MS

The samples were analysed on an Agilent 1290 Infinity LC system coupled to an Agilent 6550 accurate mass Q-TOF mass spectrometer using Dual Agilent Jet Stream Electrospray Ionization (Agilent Technologies). The injection volume was 4 µl and chromatographic separation was achieved using a Zorbax SB-Aq 1.8 µM 2.1 × 50 mm column with a Zorbax SB-C8 guard column and rapid resolution cartridge (2.1 × 30 mm 3.5 μm). Elution was achieved using a linear gradient at a flow rate of 0.6 ml min^−1^ starting with 2% mobile phase B (0.2% v/v acetic acid in methanol) gradually changing to 98% eluent B over 13 min. This is followed by 1.5-min isocratic flow of 2% mobile phase A (0.2% v/v acetic acid in MilliQ water) and 1-min equilibration at 2% eluent B. Data acquisition was carried out using electrospray ionization in the positive and negative mode using full-scan analysis over a mass range of *m*/*z* 50 to *m*/*z* 1,700 in 2-GHz extended dynamic range acquisition mode. For online mass axis correction, purine and hexakis (1H,1H,3H-tetrafluoropropoxy)phosphazine (HP-0921; Agilent Technologies) were added to the mobile phase. Electrospray settings were as follows: ion spray voltage, 3.5 kV negative mode and 4 kV positive mode; capillary temperature, 325 °C; drying gas flow, 10 l min^−1^; and 45 p.s.i. nebulizer pressure.

### Untargeted metabolite profiling using FIA–TOF–MS

Untargeted metabolomics measurements were performed using FIA–TOF–MS with a setup consisting of an Agilent Series 1100 LC pump coupled to a Gerstel MPS2 autosampler and an Agilent 6550 accurate mass Q-TOF mass spectrometer using Dual Agilent Jet Stream Electrospray Ionization (Agilent Technologies), operated in negative ionization mode. The flow rate was 150 µl min^−1^ of mobile phase consisting of isopropanol/MilliQ water (60:40 v/v) buffered with 1 mM ammonium fluoride. Furthermore, 1 mM of 3-amino-1-propanesulfonic acid and 1 µM of hexakis phosphazine (HP-0921; Agilent Technologies) were added to the mobile phase as online mass axis calibration compounds. The injection volume was 5 µl, measurements were performed randomized and in double injections. Mass spectra were recorded from *m*/*z* 50 to *m*/*z* 1,700 with a frequency of 1.4 spectra per s for 0.48 min using 4-GHz high-resolution settings. Electrospray settings were as follows: ion spray voltage, 3.5 kV negative mode; capillary temperature, 325 ˚C; drying gas flow, 5 l min^−1^; and 30 p.s.i. nebulizer pressure. Fragmentor, skimmer and octopole voltages were set to 175, 65 and 750 V, respectively.

### Preparation of standards for calibration

Stock solutions of 138 purified single compounds obtained from Sigma-Aldrich were prepared by dissolving them separately in either MilliQ water, ethanol–water or methanol–water mixtures, or dimethylsulfoxide and subsequently mixed to 600 µM (for a full list of all standards see Supplementary Table 1). The mixture was aliquoted and dried at 0.12 mbar to complete dryness in a SpeedVac setup (Christ) and stored at −80 °C. Working solutions were prepared in MilliQ water with 0.4% (v/v) acetic acid and filtered before use.

Calibration curves were obtained from a 24-point serial dilution series spanning seven orders of magnitude (from 17.9 pM to 150 µM) in MilliQ water and, to assess matrix effects, in a pooled study sample background. For that, the diluted matrix was spiked with the serially diluted standard mixture. Standards were measured at the beginning and end of the measurement in a positive and negative ionization mode.

### Quantification

Linear regression of log-transformed ion counts versus log-transformed concentrations was performed on measurements from the standard dilution series, allowing for the iterative removal of up to six dilution steps from the upper concentration limit and 12 data points from the lower dilutions, with the goal of maximizing the *R*^2^ value. The lower limit of quantification and upper limit of linearity were determined as the lowest and highest accepted dilution steps, respectively. Metabolite concentrations were then calculated based on the derived slopes and intercepts. Only values within the linear range were considered for further analyses. Of 138 metabolites, 128 could be reliably detected and quantified based on these criteria. For each unique metabolite we measured up to five different modifications (deprotonated anion, protonated cation, sodium adduct cation, dimer cation and dimer sodium adduct cation) and manually selected the modification based on linear fit (highest *R*^2^ value), linear range and the reliable coverage within the dataset (maximal number of detected samples). The selected modifications, the linear fits of the respective standards and the final dataset can be found in Supplementary Table 1. Metabolite concentrations are reported as $\frac{{\mathrm{{nmol}}}}{{\mathrm{{mg}}\;{\mathrm{sample}}}}$.

### Data processing, analysis and visualization

For LC–TOF–MS data, preprocessing, peak picking and annotation were performed using MassHunter Quantitative Analysis B.07.00 software (Agilent Technologies). Metabolites were classified according to MetaCyc^56^ (https://metacyc.org/). Further data analysis, statistics and visualization were performed after raw data export in MATLAB R2021b (MathWorks) using functions embedded in the Bioinformatics and Statistics toolboxes. Graphs, illustrations and plots were finalized using Illustrator (Adobe).

### 16S rRNA sequencing of intestinal samples

For five SPF mice, we sampled both mucus and luminal content separately at five sites: duodenum, jejunum, ileum, caecum and colon. Following DNA extraction according to the manufacturer’s instructions (QIAamp PowerFecal DNA Kit, Qiagen), amplicons spanning the 16S rRNA gene variable region 3 and 4 were prepared using degenerate primers 515FY and 806R according to the protocols benchmarked for the Earth Microbiome project^57–59^. The PCR-amplified amplicons were gel purified (MinElute Gel Extraction Kit, Qiagen), and Illumina sequencing adaptors and barcodes were added followed by bead-based clean up (AMPure beads, Beckman Coulter). Samples were sequenced using an Illumina MiSeq at the Functional Genomics Center Zurich. A total of 16,557,915 sequencing reads from 72 samples (median = 173,616) served as input for the inference of Amplicon Sequence Variants (ASVs) using dada2 v.1.14 (ref. ^60^). Primer sequences (515FY = GTGYCAGCMGCCGCGGTAA, 806R = GGACTACNVGGGTWTCTAAT) were removed using cutadapt v.2.8 (ref. ^61^) and only inserts that contained both primers and were at least 75 bases long were kept for downstream analysis. Next, reads were quality filtered using the *filterAndTrim* function of the dada2 R package (maxEE = 2, truncQ = 3, trimRight = (40, 40)). The *learnErrors* and *dada* functions were used to calculate sample inference using *pool* = *pseudo* as the parameter. Reads were merged using the *mergePairs* function and bimeras were removed with the *removeBimeraDenovo* function (*method* = *pooled*). The remaining ASVs were then annotated taxonomically using the IDTAXA classifier^62^ in combination with the Silva v.138 database^63^ available at http://www2.decipher.codes/Downloads.html. Samples with fewer than 1,000 reads (nine samples in total) were not considered for downstream analyses. The resulting ASV abundance table was downsampled to a common sequencing depth (6,000 reads per sample; that is, the minimum of all 63 remaining samples) to correct for differences in sequencing depth between samples using the *rrarefy* function of the vegan R package. Abundance tables at each taxonomic level were computed by adding up the abundance of ASVs belonging to the same taxa.

### Statistical analysis

Statistical analyses were performed in MATLAB R2021b (MathWorks) using functions embedded in the Bioinformatics and Statistics toolboxes. Values between two groups (for example, between different sampling sites) were compared and statistical significances calculated using paired-sample Student’s *t*-tests (implemented in the MATLAB Statistics toolbox). Generally, five male mice served as biological replicates. Experiments were not repeated. The exact number of samples used to calculate a statistical difference is indicated in each figure legend. The false discovery rate was controlled by correcting the calculated *P* values for multiple testing (Benjamini–Hochberg procedure), and corrected *P* values ≤0.05 were considered statistically significant.

### Metabolite–microbe association analysis

To predict functional abundances within the microbiome, PICRUSt2 v.2.5.1 (ref. ^38^) was used on the analysed ASVs (Supplementary Table 10), which yielded a list of Enzyme Classification numbers for each detected microorganism. The predicted microbiome metabolic network was then reconstructed from the Enzyme Classification lists and compared with the *Mus musculus* metabolic network in KEGG using the bioservices^64^ python package. Measured metabolites were mapped to either network to predict their origin. Spatial correlations across all sample sites between microorganisms and metabolites were calculated for each pair as Spearman’s coefficient using the Scipy v.1.9.3 (ref. ^65^) python package.

### Reporting summary

Further information on research design is available in the Nature Portfolio Reporting Summary linked to this article.

## Supplementary information

Reporting Summary

Supplementary Tables 1–10 Supplementary Tables 1–10 combined in a single workbook with ten separate tabs. Data include detailed information about the molecules, specific fold changes, *P* values and other information supporting the main text and figures.

Supplementary Data Processed and quantified metabolite data per sample.

## Data Availability

Raw LC–TOF–MS data have been deposited at MassIVE and are available with the accession code MSV000091478 (10.25345/C5K06XB0Q). 16S rRNA sequencing data are available under the BioProject ID PRJNA944604 from the NIH Sequence Read Archive (https://www.ncbi.nlm.nih.gov/sra/PRJNA944604). The KEGG database was used as resource in the metabolite–microbe associations. Source data are provided with this paper.

## Source data

Source Data Fig. 1 Metabolite abundance data, scores from principal component analysis (PC1 and PC2), statistical source data (*P* values and fold changes).
Source Data Fig. 2 Statistical source data (*P* values and fold changes), metabolite concentration data.
Source Data Fig. 3 Metabolite concentration data.
Source Data Fig. 4 Statistical source data (*P* values and fold changes), metabolite concentration data.
Source Data Fig. 5 Statistical source data (*P* values and fold changes), metabolite concentration data.
Source Data Fig. 6 KEGG identifiers, statistical source data, metabolite concentration data.
Source Data Extended Data Fig. 1 Metabolite abundance data, metabolite concentration data.
Source Data Extended Data Fig. 2 Metabolite concentration data.
Source Data Extended Data Fig. 3 Metabolite concentration data.
Source Data Extended Data Fig. 4 Statistical source data, metabolite concentration data.
Source Data Extended Data Fig. 5 Statistical source data (p-values and fold changes), metabolite concentration data.
Source Data Extended Data Fig. 6 Microbial abundance data.
