# Supplementary material for: Metabolic landscape of the male mouse gut identifies different niches determined by microbial activities
Source: Nat Metab. 2023 May 22;5(6):968–80. doi: 10.1038/s42255-023-00802-1 (PMC10290957; doi:10.1038/s42255-023-00802-1)
Supplement: Supplementary file 1 — Reporting Summary [file 42255_2023_802_MOESM1_ESM.pdf]

nature portfolio

Corresponding author(s): Prof. Dr. Uwe Sauer

Last updated by author(s): Mar 28, 2023

Reporting Summary

Nature Portfolio wishes to improve the reproducibility of the work that we publish. This form provides structure for consistency and transparency in reporting. For further information on Nature Portfolio policies, see our Editorial Policies and the Editorial Policy Checklist.

Statistics

For all statistical analyses, confirm that the following items are present in the figure legend, table legend, main text, or Methods section.

n/a	Confirmed	
☐	☒	The exact sample size (*n*) for each experimental group/condition, given as a discrete number and unit of measurement
☐	☒	A statement on whether measurements were taken from distinct samples or whether the same sample was measured repeatedly
☐	☒	The statistical test(s) used AND whether they are one- or two-sided<br>*Only common tests should be described solely by name; describe more complex techniques in the Methods section.*
☒	☐	A description of all covariates tested
☐	☒	A description of any assumptions or corrections, such as tests of normality and adjustment for multiple comparisons
☐	☒	A full description of the statistical parameters including central tendency (e.g. means) or other basic estimates (e.g. regression coefficient) AND variation (e.g. standard deviation) or associated estimates of uncertainty (e.g. confidence intervals)
☐	☒	For null hypothesis testing, the test statistic (e.g. *F*, *t*, *r*) with confidence intervals, effect sizes, degrees of freedom and *P* value noted<br>*Give P values as exact values whenever suitable.*
☒	☐	For Bayesian analysis, information on the choice of priors and Markov chain Monte Carlo settings
☒	☐	For hierarchical and complex designs, identification of the appropriate level for tests and full reporting of outcomes
☒	☐	Estimates of effect sizes (e.g. Cohen's *d*, Pearson's *r*), indicating how they were calculated

Our web collection on statistics for biologists contains articles on many of the points above.

Software and code

Policy information about availability of computer code

Data collection	LC-TOF-MS data was collected using the Agilent MassHunter Acquisition software. 16S rRNA sequencing data was acquired using an Illumina MiSeq.
Data analysis	LC-TOF-MS raw data processing was performed using Agilent MassHunter Quantitative Analysis B.07.00 software (Agilent Technologies, Santa Clara, USA). Metabolomics data analysis, statistical analysis and data visualization was performed in Matlab R2021b using standard functions. Sequencing data was processed using standard packages in R.

For manuscripts utilizing custom algorithms or software that are central to the research but not yet described in published literature, software must be made available to editors and reviewers. We strongly encourage code deposition in a community repository (e.g. GitHub). See the Nature Portfolio guidelines for submitting code & software for further information.

Data

Policy information about availability of data

All manuscripts must include a data availability statement. This statement should provide the following information, where applicable:

- Accession codes, unique identifiers, or web links for publicly available datasets
- A description of any restrictions on data availability
- For clinical datasets or third party data, please ensure that the statement adheres to our policy

The LC-TOF-MS metabolomics data is deposited in the MassIVE database (MSV000091478). Sequencing data is deposited at the Sequence Read Archive (SRA), NCBI, and accessible via the BioProject ID PRJNA944604. Processed and analyzed metabolomics data are available in the Supplementary Tables provided with this paper.

Human research participants

Policy information about studies involving human research participants and Sex and Gender in Research.

Reporting on sex and gender	N/A
Population characteristics	N/A
Recruitment	N/A
Ethics oversight	N/A

Note that full information on the approval of the study protocol must also be provided in the manuscript.

Field-specific reporting

Please select the one below that is the best fit for your research. If you are not sure, read the appropriate sections before making your selection.

☒ Life sciences ☐ Behavioural & social sciences ☐ Ecological, evolutionary & environmental sciences

For a reference copy of the document with all sections, see nature.com/documents/nr-reporting-summary-flat.pdf

Life sciences study design

All studies must disclose on these points even when the disclosure is negative.

Sample size	To account for individual differences between animals, 5 SPF and germ-free mice were sampled for metabolomics. Group sample sizes of ≥3 mice were determined from preliminary experiments that indicated sufficient power to discriminate repertoire effects. The 15 different sampling sites along the gut account for the longitudinal differences and were determined based on anatomical features. Intestinal contents were separated from the mucus to account for intra-site variability. For sequencing we sampled 5 sites over the length of the gut of 5 individual SPF mice and further separated contents from mucus. 5 sampling sites are the maximum that still allows for sufficient amounts for DNA preparation.
Data exclusions	Metabolites that could not be reliably detected and quantified within the linear range based on analytical standards (10 metabolites) were excluded. During sequencing data processing, samples with less than 1000 reads (nine samples in total) were not considered for downstream analyses.
Replication	Biological replicates (n=5 mice) account for differences between individuals and were determined from preliminary experiments that indicated sufficient power to discriminate repertoire effects. To be mindful of animal numbers, no replication attempts were made.
Randomization	Metabolomics samples were measured in randomized order. Sequencing samples were prepared at the same time to control variability. Amplicon PCRs were run in 2 randomized batches, so no experimental groups can be distinguished.
Blinding	The investigators were not blinded during sample preparation and data acquisition to aspects relating to microbiome status (SPF or germ-free), sampling site and intestinal habitat (mucus or content). During data acquisition, no blinding was performed but since samples were measured in a randomized order, blinding was not necessary. During data-driven analyses such as hierarchical clustering and principal component analysis, investigators were blinded to group allocation. For differential analyses, no blinding was performed.

Reporting for specific materials, systems and methods

We require information from authors about some types of materials, experimental systems and methods used in many studies. Here, indicate whether each material, system or method listed is relevant to your study. If you are not sure if a list item applies to your research, read the appropriate section before selecting a response.

Materials & experimental systems

n/a	Involved in the study
☒	☐ Antibodies
☒	☐ Eukaryotic cell lines
☒	☐ Palaeontology and archaeology
☐	☒ Animals and other organisms
☒	☐ Clinical data
☒	☐ Dual use research of concern

Methods

n/a	Involved in the study
☒	☐ ChIP-seq
☒	☐ Flow cytometry
☒	☐ MRI-based neuroimaging

Animals and other research organisms

Policy information about studies involving animals; ARRIVE guidelines recommended for reporting animal research, and Sex and Gender in Research

Laboratory animals	Colonized specific pathogen free (SPF) mice on a C57BL/6J background were purchased from Envigo. Germ-free C57BL/6J mice were generated via caesarian section and maintained with aseptic husbandry within flexible film isolators with 14h/10h light/dark cycles, 20°C and 40% humidity. All mice were maintained at the Clean Mouse Facility at the University of Bern, Switzerland. All mice were 10-14 weeks of age, male, and confirmed to be pathogen-free.
Wild animals	No wild animals were included in this study.
Reporting on sex	All mice were male. No conclusions with respect to sex effects can be made.
Field-collected samples	No field-collected samples were included in this study.
Ethics oversight	All mouse experiments were performed in accordance with Swiss Federal and Cantonal regulations. Permission was granted by the Commission for animal experimentation of the Kanton Bern.

Note that full information on the approval of the study protocol must also be provided in the manuscript.
